# Supplementary material for: Genetic Identification and Drug-Resistance Characterization of Mycobacterium tuberculosis Using a Portable Sequencing Device. A Pilot Study
Source: Antibiotics (Basel). 2020 Aug 27;9(9):548. doi: 10.3390/antibiotics9090548 (PMC7559383; doi:10.3390/antibiotics9090548)
Supplement: Supplementary file 1 [file antibiotics-09-00548-s001.pdf]

Suppl Table 1. Comparison of MinION based TB surveillance and current probe based or culture based methods.

TB surveillance	Culture free (~6 weeks)	Decentralized laboratory	Deployable pocket sized	Ultra-long genome sequencing	<24hr surveillance	16S rRNA lung microbiota
MinION based	**yes**	**yes**	**yes**	**yes**	**yes**	**yes**
Real-time probe based	**yes**	no	no	no	**yes-limited to known target loci**	no
DST based	no	no	no	no	no	no
